# Investigating silent strokes in hypertensives: a magnetic resonance imaging study (ISSYS): rationale and protocol design

**DOI:** 10.1186/1471-2377-13-130

**Published:** 2013-10-02

**Authors:** Iolanda Riba-Llena, Carmen Ioana Jarca, Xavier Mundet, Jose L Tovar, Francesc Orfila, Antonio López-Rueda, Cristina Nafría, Jose L Fernández, Xavier Castañé, Mar Domingo, José Álvarez-Sabín, Inés Fernández-Cortiñas, Olga Maisterra, Joan Montaner, Pilar Delgado

**Affiliations:** 1 Neurovascular Research Laboratory, Institut de Recerca Vall Hebron, Universitat Autònoma de Barcelona, Passeig Vall d’Hebron 119-129, Barcelona, 08035, Spain; 2 Primary Healthcare, CUAP Horta, SAP Muntanya, Barcelona, Spain; 3 Primary Healthcare University Research Institute IDIAP Jordi Gol, Barcelona, Spain; 4 Nephrology Service, Vall Hebron's General University Hospital, Barcelona, Spain; 5 Neurorradiology, Clínica Dr. Manchón, Barcelona, Spain; 6 Neurology Service, Neurovascular Section, Vall Hebron Hospital, Autonomous University of Barcelona, Barcelona, Spain

## Abstract

**Background:**

Silent brain infarcts are detected by neuroimaging in up to 20% of asymptomatic patients based on population studies. They are five times more frequent than stroke in general population, and increase significantly both with advancing age and hypertension. Moreover, they are independently associated with the risk of future stroke and cognitive decline.

Despite these numbers and the clinical consequences of silent brain infarcts, their prevalence in Mediterranean populations is not well known and their role as predictors of future cerebrovascular and cardiovascular events in hypertensive remains to be determined.

ISSYS (Investigating Silent Strokes in Hypertensives: a magnetic resonance imaging study) is an observational cross-sectional and longitudinal study aimed to: 1- determine the prevalence of silent cerebrovascular infarcts in a large cohort of 1000 hypertensives and to study their associated factors and 2-to study their relationship with the risk of future stroke and cognitive decline.

**Methods/Design:**

Cohort study in a randomly selected sample of 1000 participants, hypertensive aged 50 to 70 years old, with no history of previous stroke or dementia.

On baseline all participants will undergo a brain MRI to determine the presence of brain infarcts and other cerebrovascular lesions (brain microbleeds, white matter changes and enlarged perivascular spaces) and will be also tested to determine other than brain organ damage (heart-left ventricular hypertrophy, kidney-urine albumin to creatinine ratio, vessels-pulse wave velocity, ankle brachial index), in order to establish the contribution of other subclinical conditions to the risk of further vascular events. Several sub-studies assessing the role of 24 hour ambulatory BP monitoring and plasma or genetic biomarkers will be performed.

Follow-up will last for at least 3 years, to assess the rate of further stroke/transient ischemic attack, other cardiovascular events and cognitive decline, and their predictors.

**Discussion:**

Improving the knowledge on the frequency and determinants of these lesions in our setting might help in the future to optimize treatments or establish new preventive strategies to minimize clinical and socioeconomic consequences of stroke and cognitive decline.

## Background

Hypertension is the most important modifiable vascular risk factor for stroke and it is commonly widespread in the aging population.

For a long time, treatment for hypertension has been focused on blood pressure (BP) levels as the main measure to determine the need and type of treatment. This approach has changed over the past years, emphasizing that diagnosis and treatment should be based on the quantification of global cardiovascular risk. According to the European guidelines on hypertension [1], common variables used to stratify risk are based on the presence of vascular risk factors (family history of premature cardiovascular disease, smoking habits, glucose and lipid parameters) plus further identification of clinical or subclinical target organ damage. Hypertension-related disease in several organs might indicate progression and markedly increase the risk beyond that caused by the BP levels or risk factors presence.

Since evidences advise for different goals of treatment in high risk individuals as compared with lower risk hypertensives [1], whenever possible, it is recommended to measure target organ damage in different tissues (i.e. heart, blood vessels, kidney and brain) because multiorgan damage is associated with worse prognosis [2]. Many different markers have been already described, such as electrocardiographic/echocardiographic markers, intima media thickness, pulse wave velocity as a marker of arterial stiffness or markers of endothelial dysfunction, among others, that can be useful identifying target organ damage. The main limitations are of course, costs and availability of diagnostic procedures and particularly for subclinical conditions, there is still limited knowledge on their predictive capacity for risk evaluation.

Regarding brain as hypertension target organ, subclinical or “silent” vascular brain lesions (i.e. infarcts, microbleeds, white matter changes) are often detected by neuroimaging in asymptomatic patients. Specifically, silent brain infarcts are five times more frequent than stroke in general population, and increase significantly both with advancing age and hypertension [3]. The term “silent” might not be entirely appropriate since these lesions could be often associated with unnoticed or subtle symptoms in patients that never asked for an evaluation, making impossible a diagnosis of stroke. The prognosis associated with these “silent” infarcts is not favourable at all, and their presence independently predicts further stroke and cognitive decline [4,5]. For all these reasons, silent brain infarcts have been recently included in the AHA Updated definition of stroke, thus emphasizing their clinical relevance [6]. This will have important consequences in public health, as it is expected to largely increase stroke prevalence. Moreover, their detection might have the potential to improve the selection of patients at higher risk for future stroke and cardiovascular events, who might benefit from more aggressive preventive treatments.

Hypertension has been related not only to the presence of silent vascular brain lesions but to the appearance of new lesions on follow-up, which can occur in up to 40%, considering progression of white matter changes [7]. Interestingly, this effect on progression is much more relevant at younger or midlife patients than later on [7,8]. Therefore, preventive strategies should pay much more attention to younger subjects, who are likely to have long term exposure to an increased risk in the following years [1].

Studies focusing on hypertensive participants have been performed, and reported a prevalence of silent brain infarcts that ranges from 20 to 86% of subjects aged 40 to 88 years old. Of note, most of these studies have included mainly Japanese populations, whereas data on Mediterranean Caucasian populations is still limited [7].

With this background, the ISSYS is designed as a cross-sectional and longitudinal study aimed to: (1) investigate the prevalence of silent cerebrovascular lesions, as signatures of brain organ damage, in a cohort of middle and advanced aged (50–70 years old) caucasian Mediterranian hypertensives and (2) to study their relationship with the risk of future stroke and cognitive decline.

## Methods/Design

ISSYS (Investigating Silent Strokes in Hypertensives: a magnetic resonance imaging study) is an observational cross-sectional and longitudinal study aimed to determine the prevalence of silent cerebrovascular lesions in a large cohort of hypertensives and to study their associated factors.

On baseline all participants will be also tested to determine other than brain (vascular, kidney, heart) organ damage, in order to establish the contribution of other subclinical conditions to global vascular risk.

Follow-up is planned to last for at least 3 years, to assess the rate of further Stroke/TIA and cardiovascular events as well as cognitive impairment, and their predictors.

### Subject selection

The basic design of ISSYS is a cohort study among 87000 persons aged 50 to 70 years old and living in the district of the north metropolitan area of Barcelona (SAP Muntanya). This site was chosen for several reasons. First, in the Primary Healthcare system there is a computer-based registry for all patients in this area who mainly attend these services rather than other private options. Second, the study is coordinated between the Primary HealthCare services in this area and the researchers from Research Institute and Vall d’Hebron Hospital, which is the public health tertiary reference centre for this area.

The study is carried out in patients diagnosed of essential hypertension who are routinely attended by general practitioners. According to the registry, around 27000 participants could be eligible for the study, since they are hypertensives and stroke-free and have been randomized after stratification by age, gender and prevalence of hypertension covering all the area.

After randomization, patients have been invited by phone to participate in the study and scheduled for a baseline evaluation at their own Primary Care centre.

Estimated sample size will be 1000 participants, who will be enrolled during 18 months and then participants will be followed-up, for at least three years.

The study protocol has been approved by the Ethics Committee of Vall d’Hebron Hospital and IDIAP Jordi Gol (University Research Institute in Primary Care).

### Baseline visit and procedures

#### Inclusion and exclusion criteria

At inclusion visit, fulfilment of inclusion and exclusion criteria is re-assessed by investigators. Briefly, inclusion criteria consists on: 1)Patients with essential hypertension diagnosed at least one year earlier; 2) Age comprised between 50 and 70 years; 3) Patients who give their consent to participate in the study.

Patients are excluded when: 1) they have history of previous stroke or dementia; 2) Brain MRI is contraindicated; 3) there is a suspicion of white coat hypertension syndrome or 4) patients affected by a terminal illness preventing future follow-up examinations, based on the investigator criteria.

A particular effort is made to rule out the presence of a previous stroke, and for that purpose investigators are trained and an adaptation of the Stroke Symptom Questionnaire by Berger K and collaborators is used [9].

Likewise, when a suspicion of dementia appears, following DSM-IV-R criteria [10] the patient is not included in the study and a proper evaluation in the presence of a caregiver is therefore recommended.

#### Clinical data collection

**Figure 1:**
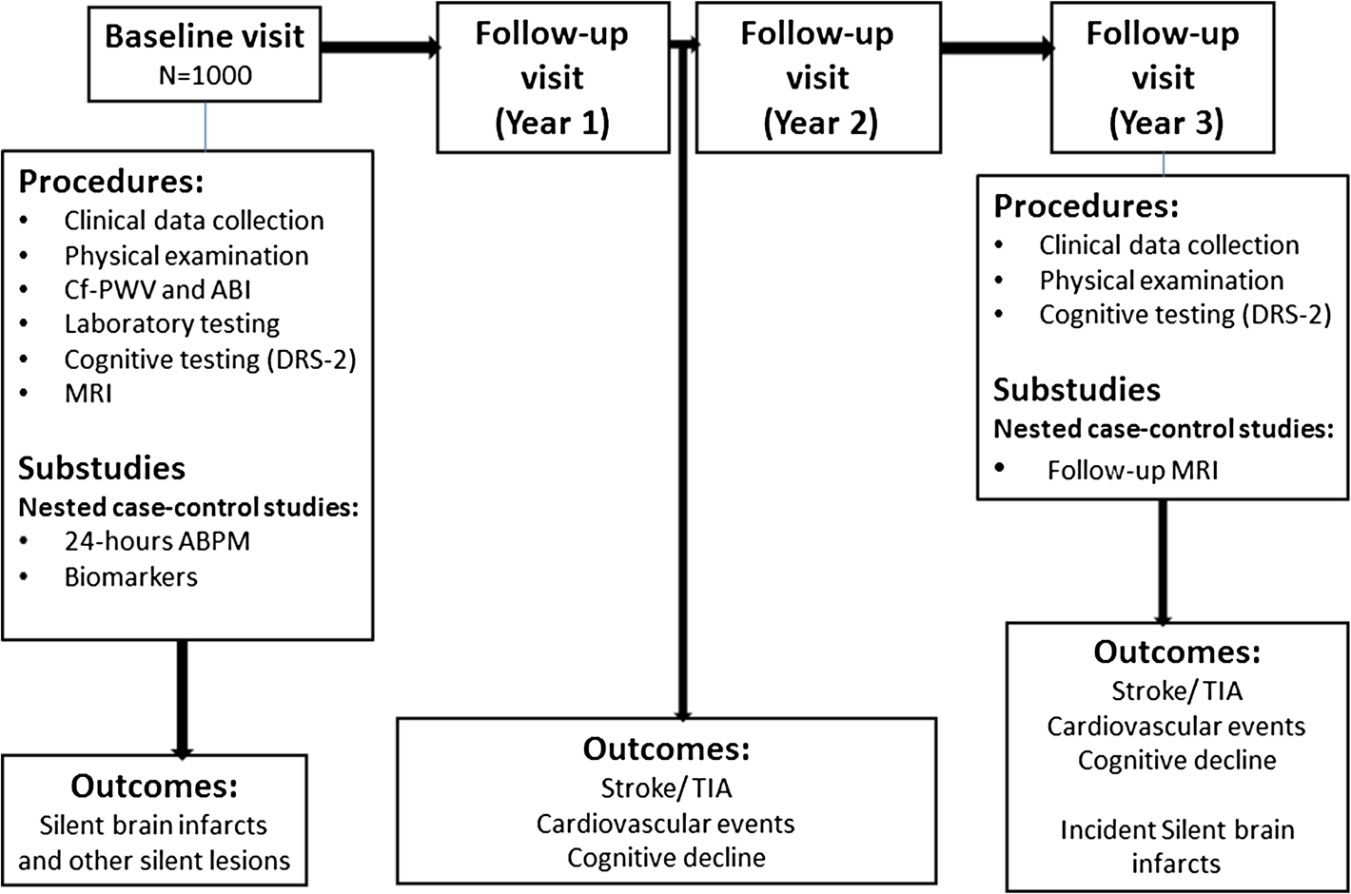
ISSYS procedures and outcomes for baseline and follow-up visits.

All procedures for baseline and follow-up visits are summarized in Figure 1.

After inclusion, the participant is asked about demographical and personal medical history. Briefly, demographical information includes age, gender, ethnicity, current or former occupation, and the maximum educational level (or completed years of schooling) achieved by the patient.

Regarding medical history, the participant is asked about the duration of hypertension, the presence of other vascular risk factors such as diabetes mellitus, hyperlipidemia, alcohol intake (grams per week), smoking habit (current, former, never) and family history of premature vascular disease and dementia in first grade relatives.

Also a directed questioning is performed to assess for the existence of an established cardiovascular, kidney or systemic disease, together with the history of retinal abnormalities or the presence of a sleep apnea syndrome. Global vascular risk is calculated applying the SCORE risk charts and Framingham-calibrated REGICOR function when appropriate [11,12].

Data concerning ambulatory and home blood pressure, home medication and treatment adherence is collected. To evaluate treatment adherence, the validated questionnaire published by Morisky and collaborators [13] is used. Briefly, this scale was developed to assess treatment compliance in hypertension and currently is used in many other chronic conditions. It consists in 4 questions, with yes/no answers which should be asked along the clinical interview, and reflect the patient’s attitude towards treatment. It is useful to find out whether or not the patient is a good complier and the causes for non-adherence.

Finally, the clinical interview ends with two self-administered questionnaires assessing life-style habits (physical activity and diet). Physical activity is evaluated by means of the International Physical Activity Questionnaire (IPAQ) [14] and dietetic habits are evaluated with a short questionnaire on frequency of dietary intake [15].

#### Physical examination and vascular testing

Regarding physical examination, some measures are taken and recorded such as height, weight, waist circumference and BP (mean of the last two out of three measurements after five minutes rest).

Plus, a standard 12-lead ECG is performed to assess for signs of left ventricular hypertrophy (single measurement of R wave in aVL [16]) and heart rhythm disorders.

Afterwards, vascular testing is performed with the Vicorder™ device (Skidmore Medical Ltd, Bristol, UK). The Vicorder™ is small, portable, non-invasive and non-operator dependent device suited for use in community based studies [17]. This system provides two BP measurement channels and two Photoplethysmography (PPG) channels for the measurement of blood flow.

Briefly, two measurements are taken for each patient: carotid-femoral pulse wave velocity (cf-PWV) and the ankle-brachial index (ABI).

Cf-PWV is measured as the best approximation of aortic pulse wave velocity (aPWV), a marker of arterial stiffness. For the measurement, the patient should be resting in a supine position, with the head and shoulders raised by about 30 degrees allowing venous return from the brain and avoiding signal contamination by the Jugular vein. A neck-pad should be placed at the lower centre part of the Right Common Carotid Artery as tightened as possible without discomforting the patient. A distal BP cuff should be located on the ipsilateral upper thigh, as high to the groin as possible. The neck-pad and thigh cuff are inflated by the Vicorder to 60 mmHg and then deflated to obtain a pressure tracing. Cf-PWV is calculated by the Vicorder by comparing carotid and femoral pressure tracings after a stable pattern is obtained. Then, cf-PWV is defined as the ratio of the distance between the carotid and the thigh position and the time it takes for the pulse wave to travel from the proximal to the distal locations. For anatomical reasons, the distance between suprasternal notch and the centre of femoral cuff is chosen as the best estimation of the distance between the two arterial sites.

ABI is measured according to the current guidelines of the American Heart Association for each side as the ratio of the highest systolic BP of each ankle and the highest systolic BP of both upper limbs [18]. The lowest of the right and left ABI values will be used.

In this case, systolic BP is determined by PPG, and as it has been previously described [19]. PPG is a fast and accurate technique and can be considered a good alternative to Doppler ABI measurement. Both sides can be examined simultaneously. Briefly, a cuff is placed around the limb of interest and a PPG signal is obtained distal to the cuff. Then, the cuff is inflated to a pre-defined target pressure and afterwards the cuff will automatically bleed pressure and the PPG signal reappears when the systolic BP is reached.

#### Cognitive assessment

On baseline, all patients will be evaluated by means of the Dementia Rating Scale-2 developed by Mattis, which is a screening tool for dementia and mild cognitive impairment. Our complete cognitive assessment protocol on baseline and follow-up has been published in detail previously [20].

#### Laboratory testing

A blood sample will be drawn after overnight fast where the basic hematology (hemoglobine, leukocyte and platelet count) and biochemistry profile (glucose, total cholesterol, creatinine, sodium, potassium and liver function) are determined. Also, plasma and serum will be obtained on baseline visit after 15 minutes centrifugation (3500 rpm) and frozen at -80°C for future biomarker determination. DNA and RNA will be also obtained and stored for further studies.

Finally, a urine sample will be collected and sent to central laboratory for albumin to creatinine ratio (UACR) determination.

#### Neuroimaging protocol

A brain MRI with a pre-stablish data acquisition protocol (Table 1) will be performed within the next month after study entry. All examinations will be performed with the same 1.5 Tesla MR (Signa HDx 1.5, General Electrics, Waukeska, WI). MR will include axial and sagittal T1 weighted images. Midline sagittal images will be used to identify the anterior-posterior commissure line, along which all oblique axial images are aligned. Also, Axial Propeller T2-weighted images, axial fluid-attenuated inversion recovery (FLAIR) and axial GRE images will be obtained. Images will be displayed on workstations monitors to be evaluated by trained readers blinded to patients’ characteristics. All images will be primarily assessed by two neuroradiologists and in a second term by the same readers plus an experienced stroke neurologist. Intra and inter-reader concordance will be provided for all lesions of interest and disagreements in assessment will be solved by consensus.

**Table 1:** MRI parameters in the ISSYS

**Sequence**	**Mode**	**Time**	**TR/TE**	**TI**	**Number of slices**	**Slice thickness/gap (mm)**	**FOV**	**Matrix**
Localizer	3D							
SE SAG T1-w	2D	3:06	400/10		20	5/1.5	256	256x224
SE AX T1-w	2D	2:58	520/10		24	5/1.0	256	256x224
Propeller AX T2-w	2D	2:40	5500/125		20	5/1.5	256	448
AX FLAIR	2D	3:20	10000/120	2200	20	5/1.5	256	320x192
AX GRE	2D	1:55	675/18		20	5/1.5	256	288x224

*SE* Spin Echo, *SAG* Sagital, *AX* Axial, *Propeller* Periodically Rotated Overlapping Parallel Lines with Enhanced Reconstruction, *FLAIR* Fluid Liquid Attenuated Inversion Recovery, *GRE* Gradient Echo, *TR* Repetition Time, *TE* Echo Time, *FOV* field of view, Matrix (Frequency x Phase).

Silent brain infarcts will be defined as previously [21] as lesions of ≥ 3 mm in diameter in their widest dimension, with cerebrospinal fluid signal characteristics in all pulse sequences, and with a hyperintense rim surrounding the lesion in FLAIR images. A particular effort will be made to differentiate these cavitated lacunes from large dilated perivascular spaces for lesions >3 mm, based on location criteria. Lesions located in areas with high prevalence of enlarged perivascular spaces, such as the lower third of basal ganglia will not be considered as infarcts. Anatomical localization for infarcts will be recorded as cortical, subcortical, basal ganglia, brainstem and cerebellum and number of lesions will be counted in case of multiple lesions.

An additional analysis will be performed for lacunar infarcts, defined as those of minimum 3 mm diameter and maximum 20 mm, located at the basal ganglia, internal capsules, thalamus, deep cerebral white matter and brainstem. An infarct located in the cortex, even if it reaches the subcortex will be recorded as cortical.

White matter hyperintensities will be rated according to the Age-related white matter changes (ARWMC) scale developed by Wahlund and col [22] that assesses presence and severity of white matter changes in the frontal, parietooccipital, temporal, basal ganglia and infratentorial areas separately in each hemisphere, ranging from 0 to 30 points.

Enlarged perivascular spaces (EPVs) or Virchow-Robin spaces will be defined as small (<3 mm), sharply delineated structures of cerebrospinal fluid (CSF) intensity following the course of perforating vessels and will be rated in T2-weighted images at the centrum semiovale, basal ganglia and midbrain following the scale reported by Doubal and collaborators [23]. Briefly, for basal ganglia and centrum semiovale, the rating will be as follows: 0: Absent; 1: Mild (from 1 to 10 EPVs); 2: Moderate (from 11 to 20 EPVs); 3: Frequent (from 21 to 40 EPVs); 4: Severe (more than 40 EPVs). For midbrain a rating of 0 will be considered when no EPVs is visible, and a rating of 1 when they are visible. For rating purposes, both hemispheres will be considered separately and the highest score of them will be chosen.

The presence of brain microbleeds, together with their number and location will be recorded following the Brain Observer Microbleed Scale (BOMBS) scale developed by Cordonnier and collaborators [24].

**Figure 2:**
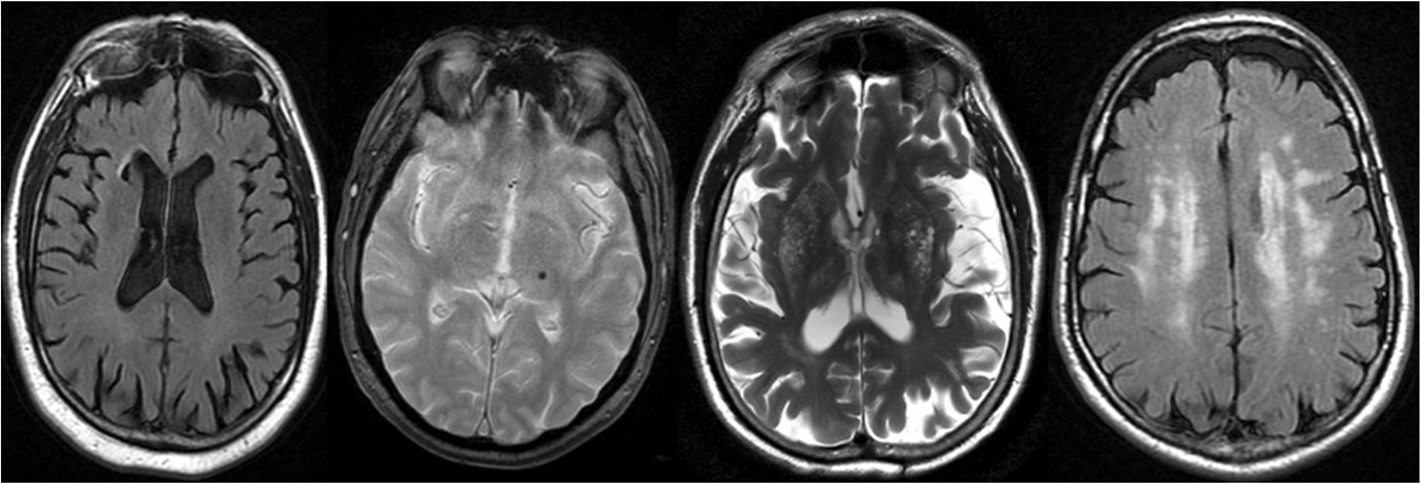
**Representative examples of subclinical/silent cerebrovascular lesions.** From left to right: Brain infarct affecting caudate nuclei (FLAIR MRI), brain microbleed in left thalamus (GRE MRI), enlarged perivascular spaces involving basal ganglia (T2 MRI) and extensive white matter changes (FLAIR MRI).

Representative MRI images for the lesions of interest are shown at Figure 2.

### Follow-up visits

The participants will be contacted yearly by phone, one and two years after the inclusion visit. They will be asked about BP control, appearance of new vascular risk factors, and the presence of stroke/TIA or new vascular events. In case of any event has occurred, this will be verified by checking clinical records from their family doctors and hospital records. Participants will also be asked about medical treatment adherence using the same questionnaire performed at baseline and treatment adverse events.

Finally, three years after inclusion a new visit is planned by the investigator’s team at the primary care center where the patient belongs, when data relative to new medical history and physical examination (blood pressure, height and weight and waist circumference measurement) will be collected, together with a new cognitive assessment with the Dementia Rating Scale-2.

#### Preventive treatments during follow-up

After baseline visit is completed and MRI is performed, primary care physicians will receive a summary with the most relevant results for each participant. Family doctors will take care of vascular risk factor control, following their routine practice. As for silent brain infarcts, since at present there is no clear evidence of whether a secondary stroke prevention should be applied to these patients and until new information comes from randomized clinical trials, recommendations will be to optimize BP control in all cases as much as possible and considering start antiplatelet treatment and/or cholesterol-lowering drugs in cases with the estimated global vascular risk is high (≥10 score in the Framingham-calibrated REGICOR function) [1].

### Study outcome

The primary outcomes of this study are the determination of the prevalence of silent brain infarctions and other silent lesions (as described in the neuroimaging protocol) and the presence and time to first-ever stroke (fatal or non-fatal ischemic or hemorrhagic stroke) or TIA. Stroke will be defined clinically as a focal neurological deficit thought of vascular origin, lasting more than 24 hours and confirmed by clinical evaluation and the use of a brain CT scan or MRI. TIA will be defined according to the classical definition, as an acute focal neurologic deficit due to cerebral ischemia that resolves completely within 24 hours, regardless of neuroimaging findings.

Other cardiovascular events will be analysed as secondary events: coronary events (myocardial infarction, angina requiring hospitalization, coronary angioplasty or surgery); cardiac failure requiring admission to hospital; vascular complications (lower limbs, aorta or carotid arteries) requiring revascularization and vascular death.

Finally, cognitive decline will be evaluated as secondary end-point during follow-up.

### Substudies (case–control studies nested in the ISSYS cohort)

#### Twenty-four hour ambulatory blood pressure monitoring (ABPM)

A nested case–control study within the ISSYS cohort will be performed with 24-hour ambulatory BP monitoring. Cases will be considered as participants in whom silent brain infarctions are detected versus controls (participants with no brain infarctions). Cases will be matched with controls by age, gender, other vascular risk factors and antihypertensive treatment in a ratio of 1 to 2/ 1 to 3.

Oscillometric ABPM measurements will be obtained using a Spacelabs 90217-5Q device (Spacelabs Healthcare, Issaquah, Washington, USA), validated according to the protocol of the British Hypertension Society [25]. The BP measurements will be made every 20 minutes during daytime and every 30 minutes during sleeptime in a day of standard activity and with a cuff suited to the size of the patient’s arm. All recordings with at least 70% of valid readings or at least 45 measurements will be considered for the analysis.

Daytime ambulatory hypertension will be defined as a mean daytime BP ≥ 135/85 mmHg and sleeptime ambulatory hypertension as a mean BP ≥ 120/70 mmHg, according to the ESH Guidelines(1).

Circadian BP patterns will be assessed, considering a nondipping status as a night to day ratio of mean systolic blood pressure (SBP) of 0.9 or more. Also the sleep-through morning surge defined as the morning BP (2-hour average of four 30-minute BP readings just after wake-up) minus the lowest nocturnal BP (1-hour average of the 3 BP readings centered on the lowest night-time reading) will be calculated [26].

#### Biomarkers determination

Also, substudies on protein and genetic biomarkers will be performed following the same strategy of a case–control study nested in this cohort.

#### Follow-up neuroimaging studies

A follow-up MRI is planned after the third year of follow-up for a nested cohort of cases and controls according to the presence of silent infarcts at baseline. This will allow us to describe the incidence of new lesions.

### Data entry and monitoring

Data will be collected by investigators and supporting staff and transferred to an electronic case report form (eCRF) on a weekly basis. No personal data enabling the identification of the participants will be included in the eCRF. Previous training is warranted for all investigators before access to the live database. Data are secured from external violation by limiting access to the computer system by individual user name and password protection.

In order to minimize missing or wrong data, external monitoring for the main outcome variables is planned, including clinical and radiological variables for at least 10% of randomly selected participants.

### Statistical methods

Sample size calculation representative for our population concerning prevalence of silent infarcts has been estimated using data of population-based studies published before, which reported specific data on silent brain infarcts prevalence in participants aged 50 to 70 years old [27-30]. According to them, the expected prevalence should be about 10%. Therefore, after applying the Ene 2.0 software, with a confidence interval of 95% and an accuracy of 2%, sample size should be of at least 865 individuals, which was increased to 1000 individuals by taking into account possible losses.

Statistical analysis will be performed with the SPSS 15.0 statistical package (Chicago, Ill., USA). Statistical significance for intergroup differences will be assessed by the χ^2^ or Fisher’s exact test for categorical variables and by the T-test, ANOVA, Mann–Whitney U and Kruskal-Wallis test for continuous variables. The correlations between continuous variables will be determined with Spearman’s or Pearson’s coefficients, as appropriate. A p value <0.05 will be considered significant. Logistic regression models will be performed to identify potential predictors of silent brain infarcts and other silent lesions. Finally, Cox proportional hazards multivariate analysis will be used to identify clinical predictors of stroke/TIA or further CVE, adjusted by variables showing p values <0.1 on univariate testing. Results will be shown as OR or HR, as appropriate, with their corresponding 95% confidence intervals.

## Discussion

Silent brain infarcts have been recently included into the new definition of stroke, given that their high prevalence and clinical consequences, such as further strokes and cognitive decline do not support to treat them as innocent findings anymore.

Although it is known that silent infarcts are associated with age and vascular risk factors, particularly hypertension, there is limited information on the prevalence of this condition in our setting. Studies restricted to hipertensives participants have been performed mainly in Asian populations and they are mostly cross-sectional, with no prospective follow-up to address either the incidence of new lesions on imaging or the presence of future strokes or cognitive decline.

ISSYS is designed to investigate both the prevalence of silent brain infarcts and the incidence of strokes, cognitive decline and appearance of new brain infarcts after three years of follow-up in a cohort of 1000 Mediterranian hypertensives.

Moreover, we will study the risk factors associated with their presence and the relationship between them and other hypertensive target organ damage, such as those occurring in heart, kidney or vessels, in order to know their single and combined contribution to the global vascular risk in each patient.

Hopefully, the better knowledge on the frequency and determinants of these lesions will help in the future to optimize treatments or establish new preventive strategies to minimize clinical and socioeconomic consequences of stroke and dementia.

## Consent

Written informed consent was obtained from the patient for the publication of this report and any acompanying images.

## Competing interests

The authors declare that they have no competing interests.

## Authors’ contributions

I R-L has drafted the first version of this manuscript and contributed to design, will carry out baseline visits and will participate in analysis and interpretation. CJ will carry out baseline visits and coordination with Primary Healthcare. XM and FO contributed to protocol design, randomization and selection of participants and coordination with Primary Healthcare. JT has participated in the design and provided expertise in ambulatory BP monitoring substudies. A L-R and JL F contributed to neuroimaging protocol design and will carry out MRI imaging readings. CN and I F-C have participated in the subjects’ enrolment and will perform phone follow-up visits and cognitive testing. XC has contributed to design of vascular studies and will participate in baseline visits. MD has contributed to acquisition and interpretation of electrocardiographic studies and will participate in baseline visits. J A-S and OM have contributed to design and coordination with other hospital departments. JM is the director of the Neurovascular Research Lab and has contributed to conception, design, revised critically this article for intellectual content and will provide support for the study development. PD has conceived and designed the study and is the principal investigator. All authors have read and approved the final manuscript.

## Pre-publication history

The pre-publication history for this paper can be accessed here:

http://www.biomedcentral.com/1471-2377/13/130/prepub
